# The Genetic and Environmental Factors for Keratoconus

**DOI:** 10.1155/2015/795738

**Published:** 2015-05-17

**Authors:** Ariela Gordon-Shaag, Michel Millodot, Einat Shneor, Yutao Liu

**Affiliations:** ^1^Department of Optometry and Vision Science, Hadassah Academic College, 9101001 Jerusalem, Israel; ^2^School of Optometry, The Hong Kong Polytechnic University, Hung Hom, Hong Kong; ^3^Department of Cellular Biology and Anatomy, The Medical College of Georgia, Georgia Regents University, Augusta, GA 30912, USA

## Abstract

Keratoconus (KC) is the most common cornea ectatic disorder. It is characterized by a cone-shaped thin cornea leading to myopia, irregular astigmatism, and vision impairment. It affects all ethnic groups and both genders. Both environmental and genetic factors may contribute to its pathogenesis. This review is to summarize the current research development in KC epidemiology and genetic etiology. Environmental factors include but are not limited to eye rubbing, atopy, sun exposure, and geography. Genetic discoveries have been reviewed with evidence from family-based linkage analysis and fine mapping in linkage region, genome-wide association studies, and candidate genes analyses. A number of genes have been discovered at a relatively rapid pace. The detailed molecular mechanism underlying KC pathogenesis will significantly advance our understanding of KC and promote the development of potential therapies.

## 1. Introduction

Keratoconus (KC), a term which comes from the Greek words* keras* (cornea) and* konos* (cone), was first described in the literature in 1854 (Nottingham). Yet its etiology, which is multifactorial with genetic and environmental influences, remains elusive [1]. It is a corneal disorder in which the central portion of the cornea becomes thinner and bulges forward in a cone-shaped fashion resulting in myopia, irregular astigmatism, and eventually visual impairment. Until some years ago, the definition of KC included the notion of a noninflammatory process [2, 3]. However, recent evidence of overexpression of inflammatory mediators such as cytokines and interleukin 6 (IL-6) in tears of KC patients and in subclinical KC may refute this concept [4, 5] and inflammation is currently considered by some researchers to play a role in the pathogenesis of KC [1, 6] (reviewed in [7]). Further evidence comes from the reduced levels of superoxide dismutase [8] in KC whose function is to remove reactive oxygen species known to be associated with inflammatory reactions.

### 1.1. Signs and Symptoms

The onset of the disease usually occurs in the second decade of life, although some cases may develop in early adulthood [3]. It is a progressive condition which usually stabilizes by the fourth decade of life [2, 9, 10]. Early in the disease, the patient is typically asymptomatic. As the disease progresses, visual acuity decreases and eventually the patient notices visual distortion with significant vision loss. These changes are due to the development of irregular astigmatism, myopia, and in many cases corneal scarring. In addition, the cornea becomes thinner [11, 12] and less touch-sensitive [13, 14]. The disease is bilateral, although asymmetrical [3]. Initially it is often unilateral, the prevalence of which ranges from 14.3% to 41% [2, 15, 16] when detected by keratometry alone. With computerized topography the prevalence of unilaterality is greatly diminished from 0.5% to 4% [17–22]. However, the majority of patients eventually develop bilateral KC. In one study it was shown that 50% of the nonaffected fellow eyes developed the disease within 16 years [23].

KC affects both men and women. However, it remains unclear whether men or women have higher prevalence of KC. The majority of recent papers published after 1970s [13, 24–32] indicate a preponderance of men over women with KC while other studies published prior to 1970s and two recent studies reported the opposite [16, 33, 34]. In a retrospective study conducted in Netherlands [35], using data relating to over 100,000 contact lens wearers obtained from four university clinics and five noncontact lens centers between the years 1950 and 1986, the ratio of men to women was 0.5. In cases diagnosed in the period from 1950 to 1954, it remained less than 1.0 until 1970s when the number of male patients significantly increased while the number of female patients remained virtually unchanged. The ratio of KC affected men compared to women reached 1.58 for patients diagnosed in 1985 and 1986 and this difference was made more manifest with the advent of corneal topography. Several reasons may account for this observation. First, this study is based on clinics instead of population or community. Second, possible sample errors could affect the study result. Third, different technologies to diagnose KC may be used throughout the study period. Finally, hormonal differences have been invoked and it has been noted that keratoconus develops earlier and progresses more rapidly in men than women [36], which could account for its higher prevalence.

Early* biomicroscopic* signs include Fleischer's ring, which is a partial or complete circle of iron deposition in the epithelium surrounding the base of the cornea and Vogt's striae, which are fine vertical lines produced by compression of Descemet's membrane [37]. As the disease progresses, a Munson's sign, a V-shaped deformation of the lower lid, becomes noticeable as the eye looks in the downward position, as well as a bright reflection of the nasal area of the limbus called Rizzuti's sign [37]. Less common are breaks in Descemet's membrane known as hydrops, which cause stromal edema, vision loss, and associated pain [38, 39]. For patients who wear contact lenses, corneal scarring is a very common feature [40].

### 1.2. Diagnosis

Since KC is typically characterized by the progression of irregular astigmatism, thinner cornea, and increased steepening of corneal curvature, KC is often first detected in the course of an eye examination and patients may be unaware of it, even though they complain of poor vision and have sought ocular care [27, 41]. The practitioner may note a suspicious reduction in visual acuity, scissors movements in retinoscopy, distortion of keratometric images, smaller values of pachymetric corneal thickness, which often precedes ectasia, or some of the known signs of the disease during the slit-lamp examination.

The most sensitive method of detecting and confirming a diagnosis of KC is unequivocally corneal topography based on the principles of Placido disc and Scheimpflug imaging, the latter being the most sensitive method of assessing corneal shape. Topography has become the gold standard method to diagnose and monitor KC [3, 42]. It allows the early detection of subclinical cases, also called* forme fruste* or KC suspect, as well as grading the severity of the disease by producing a color-coded topographic map of the corneal surface and various indices. Several quantitative methods based on these indices have been developed. The most common are the KC prediction index (KPI), which is derived from eight quantitative indices and the KC Index (KCI %) itself derived from the KPI and four other indices [42], and the KISA % which is based on keratometric value, inferior-superior asymmetry (I-S), asymmetric bow-tie astigmatism (AST), and skewed radial axis (SRAX) values [43]. Instruments that are based on Scheimpflug imaging [44, 45] are especially important in light of recent studies that suggest that KC starts from the posterior cornea and that posterior curvature may be the best way of identifying early KC [46–49]. Pellucid marginal degeneration is easily distinguished from KC by slit-lamp examination and by a distinct videokeratographic pattern [3].

The measurement of corneal thickness made by optical coherence tomography (OCT) has been shown to be as sensitive and as specific as the topographic KISA index [12]. Other attempts at detecting KC have been made with corneal aberrometers [50], since the keratoconic corneas display a large amount of higher order aberrations, especially vertical coma. However, in a study comparing the aberrations to the inferior-superior topographic values, the latter was proved to be just as good as a detector of KC [51].

### 1.3. Treatment

A number of different treatments are used to correct the vision caused by KC. In the early stages, the condition is usually well managed by spectacles. As the condition progresses to a mild or moderate stage with irregular astigmatism, the treatment of choice is contact lenses, especially rigid gas permeable lenses. However, about 20% of patients with advanced or severe KC cannot tolerate or improve their vision sufficiently with contact lenses and will eventually need surgery. The traditional surgical intervention has been penetrating keratoplasty in which the entire thickness of the cornea is excised and replaced by a donor cornea. This operation has yielded better vision than the partial removal of a superficial corneal layer (called lamellar keratoplasty) [52] but it caused more graft rejection [53]. Recently, a technique called collagen cross-linking (CXL) has been introduced and it has been proven to be successful not only at improving visual acuity but also at stiffening thus arresting and, even in many cases, regressing the progression of KC by preventing enzymatic degradation of stromal collagen [54–57]. Further research with KC will significantly improve our understanding and therefore potential therapy for KC.

## 2. Prevalence of KC

The burden of a disease in a community is evaluated by the knowledge of how widespread is that disease. This is demonstrated by its* prevalence*, which is a proportion (or percentage) of the total number of cases at a period in time divided by the size of the population from which the cases have been determined. Another measure of burden of disease is* incidence*, which is the number of new cases presenting during a defined period of time divided by the population size from which the cases have been determined and existing during that same period of time. Moreover, if the disease is chronic, then prevalence = incidence × duration. However, these measures of disease occurrence are used to characterize the KC population at risk of the disease. In particular, it is aimed at identifying the KC population at risk (e.g., gender, age, parental consanguinity, and associated factors), the geographic location of greater occurrence, and the time when disease occurs most frequently (e.g., exposure to a risk factor and introduction of computer topography).

### 2.1. Hospital/Clinic Based Reports

The majority of prevalence studies have been conducted in a hospital clinic because of the ease of collecting data. Although these findings offer an estimate of prevalence, they are likely to underestimate the true prevalence of the disease, as patients presenting in hospitals are usually symptomatic and early forms of the condition are thus missed. In addition, these studies neglect the number of patients treated by independent optometrists and ophthalmologists. They do not take into consideration an ascertainment bias in access to health care. Although these studies are commonly cited, they must be interpreted with caution.

Until a few years ago most publications on KC referred almost exclusively to one prevalence value obtained in Minnesota, USA, in 1986 which had been found to be 0.054% (54 persons out of 100,000 people) [15]. The diagnosis was based on a mixture of scissors movements in retinoscopy and keratometry, as were the majority of prevalence studies published prior to 2011. Nevertheless, this figure was similar to those reported in Finland [59] or Denmark [62] but much higher than those reported in the Urals, Russia, at 0.0004% [60] or 0.0068% in Skope, Macedonia [63]. Still, it must be noted that the more precise videokeratography is likely to yield higher prevalence than the older methodology. Indeed, recent studies using this method report higher prevalence or incidence [27, 41, 64, 67–71], but other factors may confound a possible correlation with the method used, since they come principally from the Middle East and India with different climates and ethnic groups than Europe or North America, even if merely diagnosed with a keratometer [33].  Table 1 presents the epidemiological studies conducted in a hospital/clinic. Comments on the ethnic differences will be discussed in Section 3.2.4.

### 2.2. Population-Based Studies

Cross-sectional studies typically enroll people who volunteer to participate in the investigation, even though the population selected may represent a broad socioeconomic spectrum. Nevertheless, a selection bias may occur, since individuals with the disease may refrain from participating. On the other hand, others with visual problems may be keen to volunteer. However, the majority of volunteers are likely to have felt no particular bias. Selection bias is unlikely to cause a significant error because in some studies it was observed that a certain proportion of the volunteers who had been totally unaware of their condition were discovered to have the disease during the survey [27, 41]. Therefore, population-based screening studies are the best methodology to assess the true prevalence of the disease.

Modern videokeratography is the best method to screen subjects in a population-based study. However, for the purpose of completeness we will also mention studies using less reliable methodology. The first cross-sectional survey was carried out in 1957 at the Indiana State Fair in Indianapolis over a period of 10 days by 25 different optometrists, using a Placido disc [65]. 13,345 people were thus examined and 50 individuals exhibited a doubtful or definite keratoconic pattern, thereby indicating a prevalence of 0.37% for doubtful and definite types and only 0.12% for definite keratoconic patterns. The possible discrepancy in subjective assessment of the corneal pattern through a Placido disc, an inadequate method, by the large number of examiners rendered this study unreliable. The Central India Eye and Medical Study is a population-based study that included 4,667 subjects in rural India [33] and found a prevalence of 2.3**%**. KC was defined as an anterior corneal refractive power exceeding 48 D, as measured by keratometry. Since keratometry measures the central corneal power, it is likely to miss some inferior cones. In addition, not all subjects with refractive power exceeding 48 D will have KC. Therefore, this estimation must be viewed with caution. The population-based Beijing Eye Study included 3468 individuals [68]. Steep cornea/KC was found to be 0.960 ± 2%, defined as an anterior corneal refractive power exceeding 48 D measured using optical low coherence reflectometry biometry of the right eyes only. These results must be interpreted with the same caution as the previous study. Another investigation of French army recruits using videokeratography arrived at a prevalence of 1.2%, but the results of the various indices were more compatible with suspect than definite cases [66].

More definite prevalence studies have been conducted since 2009 in the Middle East and Asia, using in most instances videokeratography, which afford better detection. For example, Millodot et al. [27] described how they diagnosed KC with a combination of topographic pattern, dioptric power of the corneal apex, and inferior-superior asymmetry to determine normal KC suspect and definite KC.  Table 2 illustrates the population-based studies published thus far. It can be seen from the table that in the last few years almost all prevalence studies have relied on the use of videokeratography. As shown in Table 2, these modern studies result in a higher prevalence of KC than previously thought, ranging from 0.9% to 3.3%. Comments on the ethnic and geographical differences will be discussed in Section 3.2.4.

## 3. Risk Factors for KC

### 3.1. Environmental Factors

It is commonly accepted that the etiology of KC is multifactorial combining environmental and genetic factors [1, 101–103]. Moreover, it seems that an environmental factor may be essential to act as a trigger of the condition in genetically predisposed individuals. Environmental factors, which have been recognized, are eye rubbing, atopy, and UV exposure, although the relative contribution of all these factors is currently unknown [6]. An excess of any of these environmental factors cause oxidative damage to KC corneas because of the inability of KC corneas to process reactive oxygen species (ROS), which leads to a degradation process leading ultimately to corneal thinning and loss of vision [104] due to a lack of corneal enzymes such as aldehyde dehydrogenase class 3 (ALDH3), catalase, or superoxide dismutase to remove or neutralize the ROS [105].

#### 3.1.1. Eye Rubbing

An association between eye rubbing and KC has long been described [24, 78, 81, 102, 106, 107] and accepted as a risk factor. Most authors report that about half of KC patients rub their eyes, although the percentage varies according to the study (see review in [82]). Obviously, there are some variations in this association whether the eye rubbing is gentle or vigorous [79, 108] and the usual length of rubbing in KC patients is much longer (from 10 to 180 seconds) than the typically less than 15-second duration of rubbing in allergic or infective ocular disorders [109] and less than 5 seconds in people without any eye condition [78]. Noteworthy are cases of asymmetric KC in which the most affected eye was the one which was rubbed most vigorously [78, 110, 111]. Coyle [112] reported the case of an 11-year-old boy who, at the age of 5, discovered he could stop his paroxysmal atrial tachycardia by vigorously massaging his left eye (up to 20 minutes a day). At the age of 7, his ocular examination was normal. By the age of 11, the child had developed unilateral KC in his left eye. Another case reported a patient with a history of vigorous daily ritual massaging of the left eye which had led to unilateral KC in that eye [113]. A series of cases confirm the asymmetric expression of the disease in patients who habitually rub the more affected eye [110, 111, 114].

Case-control studies provide the most convincing evidence of an association between KC and eye rubbing. The first was by Bawazeer et al. [24], who conducted a logistic regression analysis that included atopy and family history of KC and found that only eye rubbing was significantly associated with the disease, with an odd ratio (OR) of 3.98. This was confirmed in other logistic analyses [115, 116]. Nevertheless, this strong association has not been reported by all authors. Although they usually find a large percentage of KC patients who rub their eyes, the control group does as well [27, 41, 79]. The discrepancy may stem from the amount of dust in dry climates inducing frequent eye rubbing in both patients and controls, thus concealing a possible association.

Still, most authors who reviewed the pathogenesis of KC consider eye rubbing to be strongly associated with the disease [6, 104, 117]. There is mechanical trauma which could be caused by chronic eye rubbing, as well as a result of poorly fitted rigid contact lenses [104, 118, 119]. Nevertheless, this association is not necessarily causative. Indeed a fair percentage of individuals develop KC without any history of eye rubbing. It could be that abnormal rubbing habits start as KC develops and vision is impaired. However, there are a large number of patients with a history of habitual eye rubbing before the development of KC [15, 120, 121] and one is compelled to accept eye rubbing as a risk factor at least in some forms of KC in genetically susceptible people [122].

The microtrauma caused to the epithelium by rubbing KC corneas generates elevated levels of matrix metalloproteinases MMP-1 and MMP-13 [123, 124], which are secreted by epithelial and stromal cells, and inflammatory mediators including IL-6 and TNF-*α* [5, 125]. The release of these factors form part of the process that leads to KC and its progression. The processes include apoptosis of keratocytes as a result of increased levels of interleukin IL-1 with subsequent loss of stromal volume [126]. Direct experimental evidence of an association between KC and eye rubbing has been demonstrated in a group of volunteers without the disease and not wearing contact lenses who were instructed to rub their eyes in a controlled fashion for 60 seconds. Basal tears were collected before and after eye rubbing and it was found that levels of MMP-13, IL-6 and, TNF-*α* were significantly increased after rubbing. The authors concluded that persistent eye rubbing, common in KC patients, may contribute to the progression of the disease by continuous elevated levels of these protease, inflammatory mediators and protease activity [127, 128].

#### 3.1.2. Atopy

Atopy is a hypersensitivity reaction, which comprises allergy, asthma, and eczema. There are some conflicting reports of an association between KC and atopy. A positive association has been noted by many authors [80, 82, 129, 130], but others did not find a statistically significant association when compared to a control group [24, 75, 131, 132]. It should be noted that in the nonsignificant findings [75] the control group came from the general population rather than an age- and sex-matched group. The discrepancy may stem not only from different severity of the condition or methods of assessment, which is based on patients' self-report, but also from the fact that some authors did not differentiate between the effects of the hypersensitivity reaction [24, 27, 75, 131, 132], whereas others only assessed one symptom of atopy, such as allergy, but did not include asthma or eczema [78, 102] and others assessed only allergy and asthma and not eczema [80]. Using a multivariate logistic regression analysis, Bawazeer et al. [24] concluded that atopy was not significantly associated with KC but with eye rubbing. These authors suggested that atopy was only associated indirectly because the itch that it induced led to eye rubbing. Still, Kaya et al. [130] showed that people with KC and atopy had a steeper and thinner ectatic cornea than age- and sex-matched people with KC but without atopy.

Allergy, induced by pollen, dust, antibiotics, or animal fur, is often associated with KC compared to controls or the general population [11, 29, 39, 59, 78–80, 82, 115]. It is found in about a third of KC patients, but the percentage varies according to the study (see Table 3). It should be noted that most of these studies were dependent on self-reported allergies. In some of these studies the control group came from the general population [29, 39, 79, 82], but a significant association was shown in several studies, which included an age- and sex-matched group [74, 78, 80, 115, 132]. Although allergy may cause eye rubbing, it is not the only provocative factor, since a much higher percentage of patients rubbed their eyes than the percentage of patients with allergy.  Asthma and particularly eczema are reported less commonly than allergy (see Table 3) and it would appear that these reactions are less frequently reported in some of the studies conducted in the Middle East, India, and Singapore [34, 61, 67, 81, 115, 133]. This may be due to the hot and sunny climate of these countries, although Georgiou et al. [25] reported small percentages among Asian living in the UK compared to white, suggesting an ethnic difference. Table 3 presents the percentage of patients with atopic reaction in several studies.

#### 3.1.3. Sun Exposure

Ultraviolet light (UV) is a source of reactive oxygen species (ROS) and excessive exposure to sunlight leads to oxidative damage to KC corneas, in which there is a reduced amount of the enzymes including aldehyde dehydrogenase class 3 (ALDH3) and superoxide dismutase necessary to remove the ROS [104, 105]. Hence, the higher prevalence of KC in hot, sunny countries compared to Europe and North America has led to the belief that the high sun exposure in these countries accounts for the high prevalence (see Tables 1 and 2). For example, in Jerusalem where the prevalence was found to be 2.34% [27], the mean annual number of hours of sunshine is 3397 according to the “Climatological information for Jerusalem, Israel” (http://www.hko.gov.hk/wxinfo/climat/world/eng/europe/gr_tu/jerusalem_e.htm). Such weather conditions are not unlike those prevailing in Saudi Arabia [61], Lebanon [67], India [33], and Iran [64, 69–71] in contrast to Finland [59], Minnesota [15], Urals [60], Japan [28, 58], or Denmark [62]. Additional evidence comes from animal experiments in which mice exposed to UV light demonstrated a degeneration of stromal collagen and stromal thinning with a marked loss of keratocytes [134]. This last study confirmed an earlier report of UV exposure of an anaesthetized rabbit cornea, which resulted in apoptosis of cells in all layers of the cornea as well as keratocytes [135].

However, it must be noted that UV radiations might provide a beneficial effect by inducing cross-linking of corneal collagen, thus mitigating either the development or the progression of the disease [136]. Moreover, sun exposure cannot explain the discrepancy found in the English Midlands where Indians, Bangladeshi, and Pakistani have 4.4 and 7.5 times [25, 30] higher KC prevalence than whites living in the same ambient environment. And neither can it account for the 7.9% KC prevalence reported in Tehran among non-Persians (Arabs, Turks, and Kurds) compared to 2.5% prevalence of Persians [70] or the significantly steeper corneas of Indians compared to Chinese or Malays, all living in Singapore [137]. Nevertheless, it is likely that the oxidative damage caused by UV radiations combined with a genetic factor such as consanguinity precipitates or accelerates the disease process. Research is needed to elucidate the role of sun exposure in KC, possibly in the form of a case-control investigation using a validated questionnaire.

#### 3.1.4. Miscellaneous

Exposure to environmental neurotoxins such as nicotine in the form of cigarette smoking has not been found to be associated with KC, neither in a case-control study [115] nor in observational studies [33]. In fact, there may be a negative correlation between cigarette smoking and KC possibly because the by-products of smoke may lead to cross-linking of collagen in the cornea [138]. On the other hand one report from the Urals indicated more cases of KC in the urban centers with polluting industries than in the rural areas [60].

### 3.2. Socioeconomic Factors

#### 3.2.1. Age

KC onset varies between the early teenage years and young adulthood and it seldom appears after the age of 35 years [2]. In a cohort of 196 patients, 18 years was the most frequent age of onset [59] and it was 15.39 (±3.95) in another study [139]. However, most reports give the age of diagnosis, which is some years after onset because the disease is usually asymptomatic at first. The mean diagnostic age ranged from 20.0 years (±6.4) [140] to 24.05 (±8.97) [31] in most studies [29, 141–143]. Interestingly, the age of first presentation was found to be significantly younger in Asians than in white patients by 4 to 5 years in three different studies carried out in the English Midlands (22.3 ± 6.5 versus 26.5 ± 8.5 [30]; 21.5 versus 26.4 [25]; and 23.0 ± 7.0 versus 27.8 ± 8.1 [144]). Recent reports on pediatric CXL demonstrate onset at the end of the first decade of life or early in the teen years [145–148]. This leads to the notion that either the age of onset has decreased or the medical community is being more diligent in early diagnosis.

Since the disease is chronic one would expect to find at least a similar proportion of patients in older compared to in younger patients. That is not the case, especially after the age of 50 years and this has intrigued many authors [9, 149–154], although in one study the number of old KC patients was found to be substantial [155]. Most of these studies report low percentage of KC patients beyond 50 years, ranging from 7.4% [152] to 15% [39], with one exception 40% [155]. The reason may rest in the more efficient methods of diagnosis of the disease in recent years, such as videokeratography, or it may be because there are now more people with an allergy in the general population [156]. Another possibility is that KC patients have reduced longevity compared to the general population, as has been suggested by some authors [150, 152, 154] because of an associated fatal condition, for example, mitral valve prolapse [157, 158], obesity [159, 160], or obstructive sleep apnea [160, 161], although the mortality rate of a population of KC patients was not found to be significantly different than that of the general population [151]. Nevertheless, the question as to what happens to KC patients beyond the age of 50 years remains to be elucidated, possibly by comparing the corneas of older KC patients with an age-matched control group.

About 20% of KC patients will eventually require surgery, although there are wide variations in percentages among the studies, with a seemingly lower percentage in the Far East (India, China, Singapore, and Japan) than in the rest of the world (see review in Kok et al. [162]). Nevertheless, the deleterious effects of this chronic disease, in which a substantial percentage of patients will require invasive surgery and for the other patients a lifelong need for specialized contact lens fitting, represent a serious burden not only for the individual but also for the national health services of a country.

#### 3.2.2. Geographic Location

It was thought that KC affected all countries equally [3]. However, it has become obvious, especially in the past decades, that KC prevalence is not the same throughout the world, as the presently available studies can reveal (see Tables 1 and 2). Northern Europe and the Urals have low prevalence [25, 30, 59, 60, 163], as well as northern USA [15, 65]. Prevalence is also low in Japan [28, 58]. On the other hand it is relatively high in countries of the Middle East [27, 41, 61, 64, 69–71], India [33], and China [68]. The Middle East countries in particular, as well as parts of India, are characterized by hot and sunny climates with very little rain as distinct from the other countries. Could the climate influence the development of KC, especially the oxidative damage caused by excessive sun exposure to ultraviolet light [104]? Is there an inherent difference in the people, such as ethnic backgrounds, or could the very different styles of life with nutrition play a role? There is also the possibility that in these countries the disease affects more the poor people, a factor known to increase the proportion of chronic diseases [164]. These are puzzling questions that need elucidation to better understand the pathogenesis of KC.

#### 3.2.3. Parental Education

It has been suggested that there exists an association between low parental education and KC, because parental education is associated with socioeconomic status [165]. Children living in poverty are brought up in environments with air, water, and waste contamination problems [166], which are hazardous to their health. As a consequence, these children are at risk or suffer from a host of disorders, such as asthma, cancer, hyperactivity, and obesity [167]. Several investigators have reported an association between obesity and KC [159–161, 168]. Therefore, it could be inferred that there exists an association between low parental education and KC since low parental education is linked to low socioeconomic status. To the best of our knowledge there is not as yet a report of such an association.

#### 3.2.4. Ethnic Differences

Until some years ago it was assumed that KC affected all races equally [3]. However, it has now been demonstrated unequivocally that there are differences in KC prevalence among ethnic groups. It was first noted by Pearson et al. [30] who found that Asians (Indians, Bangladeshi, and Pakistani) living in the English Midlands had an incidence of the disease 4.4 times higher than in whites. This was confirmed in two other investigations also conducted in the Midlands where the difference in incidence was 7.5/1 [25] and 9.2/1 [144]. Other studies have demonstrated a difference among ethnic groups of the same country. In Iran, KC prevalence was found to be three times less in the Persian ethnic population than in the non-Persians (Arabs, Turks, and Kurds) [70]. In Singapore, steep cornea possibly reflecting KC was found to be significantly steeper in Indians than in Malays or Chinese [137]. In addition, the age of onset of the disease has been found to be generally younger in Asians than in Caucasians [25, 30, 79, 144]. The age of onset, or more specifically diagnosis, of most Asians is in the early 20s whereas it was much older in the CLEK study (*n* = 1209 patients) [39]. Differences in KC prevalence and age of onset among ethnic populations strongly suggest that genetic influences play an important role in the pathogenesis of the disease. This is discussed below.

### 3.3. Familial Factors

A large positive family history of the disease may stem from either environmental or genetic causes. It is not always clear which of the two is most influential in the pathogenesis of the disease without establishing a family pedigree. The recent data on the strong association of parental consanguinity/endogamy with KC suggests a strong genetic component to the development of KC in many studies [115].

#### 3.3.1. KC in the Family

Although the most common type of KC is sporadic [102], many studies have reported the presence of large number of familial KC. The rate ranges from 5% to 27.9% [15, 41, 59, 79, 82, 102]. In the study in which a rate of 27.9% of KC was found in at least one person in the family, it was further noted that affected first-degree relatives represented 20.5% [82]. It was much lower (3.34%) in first-degree relatives when the family history was not self-reported by the cases but determined by videokeratography [169]. This was still 15–67 times higher in those who had developed the disease than in those who did not have relatives with KC. In another study in which relatives (first-degree and others) were evaluated topographically, 14% of family members were found to have KC [170]. The discrepancy between the latter two studies may reflect a greater prevalence of KC in the general population of the second, which was conducted in Turkey whereas the other was in America. Most percentages of general family history are usually lower than 20%. Typical results of family history from large sample population of KC patients are 12.4% [11], 13.5% in the Collaborative Longitudinal Evaluation of Keratoconus (CLEK) [39], and 17.8% in another large cohort [171]. Interestingly, in the Dundee University Scottish Keratoconus Study (DUSKS) [79] the rate for Caucasians was 5% but it was 25% for the small Asian subgroup (Indian subcontinent) who participated in the study. This last result is not surprising as one would expect a higher level of positive family history in communities with a greater prevalence of KC. This was the case in several studies in which KC prevalence was high and so was family history, 23% [27], 22.9% [115], and 27.9% [82], as well as in a study involving KC patients in families with a lot of children as found in northern Finland 19% versus 9% in southern Finland, where families had few children [59]. The large variation in the percentage of family members with the disease (3.34%–27.9%) may indicate different expression of KC with different modes of inheritance [59, 101, 115, 169].

#### 3.3.2. Consanguinity

Consanguinity, the marriage between relatives, has been shown to be associated with a host of disorders: childhood mortality [172], deafness [173], sickle-cell anemia [174], hydrocephalus, postaxial polydactyly and facial clefts [175], heart disease [176], multiple sclerosis [177], tuberculosis and hepatitis B [178], preterm birth [179], and physical and mental handicap [180–182].

Over the years several authors have alluded to a possible association between KC and consanguinity [25, 101, 144, 183]. Evidence was provided by a report by van der Hoeve in 1924 [184] who presented a family pedigree in which three of the six children of a consanguineous couple had KC. In another report with suggestive evidence one in 400-Pakistani family, who came from a tradition of consanguinity and living in England, was found to have KC compared to one in 30,000 whites [185]. However, the first study to establish a significant association was performed in a hospital in east Jerusalem in which KC Arab patients and controls, age- and sex-matched, were examined and all subjects completed a questionnaire asking about their parents' relationship. It was found that children of consanguineous parents had a fourfold risk of KC compared with children of unrelated parents after adjusting for other factors, using multivariate logistic regression analysis [115], and this association was much stronger with parents married to first cousins than second cousins. This result was further confirmed in a similar study conducted with students from an Arab College in Haifa in which a fivefold (or 5,1, 95% 1.41–18.33) risk of KC in offspring of consanguineous marriages [41] was found.

As already suggested by Georgiou et al. [25] and Cozma et al. [144] the large discrepancy in the prevalence of KC between Asian, mostly of Pakistani origin, and white patients could be attributed to the tradition of consanguineous, especially first-cousin marriages. In fact, practically all countries with a high KC prevalence as noted in Tables 1 and 2 are from the Middle East and India which have a tradition of consanguinity, especially in their Muslim ethnic communities [186–189]. In Pakistan, approximately 60% of marriages are consanguineous, over 80% of which are between first cousins [190]. In Israel, population surveys have found that Israeli Arabs have a high rate of consanguinity, 42–45%, with 28% being first-cousin marriages [191]. For Israeli Jews, consanguinity is much lower ranging from 1.5 to 7.1% depending on the community, with 0.4 to 1.2% being first cousins [192]. However, endogamy is relatively common among Israeli Jews and it may play a role contributing to the high prevalence of KC in Israel [27]. The high corneal steepness found in Indians compared to Chinese or Malays all living in Singapore was also suggested to have been caused by consanguinity among the former [137].

If both parents are first cousins, they could both be carriers of a mutant allele at the same locus leading to corneal ectasia. The result of these studies points not only to a genetic component of the disease, but more specifically to an autosomal recessive inheritance. This is in contrast to other forms of KC in which many patients with a positive family history described in the literature, but mainly from western countries, present a family pedigree suggesting an autosomal dominant inheritance [101, 102]. Strong support for a genetic basis for KC comes from segregation analysis of genetic models based on 95 keratoconic families evaluated by videokeratography [169]. It appears undeniable that the genetic effect of consanguinity plays an important role in the pathogenesis of KC and is the principal factor that accounts for the differences in prevalence among ethnic groups and possibly geographic locations. It may, however, require to be combined with an environmental factor to be activated and lead to KC.

Twin studies in which there is a concordance in the topographic pattern of a monozygotic pair add evidence to a genetic contribution to KC. To date, 21 pairs have been reported, although many of these were described before the advent of videokeratography. Nevertheless, more than half of these pairs were found to be concordant [31, 59, 193–195], the others being discordant [196–198]. A study comparing dizygotic (DZ) and monozygotic (MZ) twins has been reported [142] in which significantly more concordance was found in MZ than in DZ providing further evidence of a genetic contribution to the disease.

## 4. Genetic Studies of KC

### 4.1. Traditional Linkage Studies

As discussed above, genetics plays an important role in the pathogenesis of KC. Relatives of KC patients have an elevated risk compared to those with unaffected relatives. Most of the familial KC is autosomal dominant while autosomal recessive pattern has also been suggested. Family-based linkage studies have identified at least 19 candidate genetic loci that may harbour genetic mutations for KC (Table 4) [199]. This clearly indicates the genetic heterogeneity of KC pathogenesis. Although most of these genomic loci have not been independently replicated, the chr5q21.2 region has been independently replicated in three separate studies [87–89]. Recently this region has been further confirmed with high density single nucleotide polymorphisms (SNPs) based linkage [200]. The overlapping region from these three studies strongly suggests the possibility of a common locus for KC pathogenesis. Another linkage locus chr5q32-33 reported by Bisceglia et al. was identified as suggestive linkage with KC by Li et al. [87, 89]. A suggestive linkage locus in chr14q11.2 was reported by these two studies. A linkage locus chr16q22.3-q23.1 identified by Tyynismaa et al. is very close to a suggestive linkage region identified by Bisceglia et al. [87, 98]. It should be noted that Burdon et al. reported two genomic regions chr1p36.23-36.21 and chr8q13.1-q21.11 with equal evidence of linkage (LOD score of 1.9 each) [83]. Analysis of both loci concurrently, meaning digenic inheritance of two loci, suggests a two-locus LOD score of 3.4. However, no mutations were identified in six candidate genes that were expressed in the cornea [83].

A number of efforts have been performed to identify the genetic mutations in these linkage regions. A 5 Mb genomic region on chr15q22-q25 was originally mapped in a large three-generation Northern Irish family with 18 affected individuals [94, 95]. All the affected family members had severe anterior KC and early-onset anterior polar cataract [95]. The inheritance was autosomal dominant. All genes in this 5 Mb genomic region were enriched using a custom sequence capture array from NimbleGen followed by second generation sequencing (a Genome Analyzer II from Illumina). A mutation (r. 57c>u) was identified within the seed region of* miR-184*.* miR-184* is a microRNA (miRNA), which is small regulatory strands or RNA with 19–25 nucleotides in size [94]. miRNA mostly binds to complementary sequences in the 3′ untranslated region (UTR) of mRNA of target genes, leading to mRNA degradation or translational repression.* miR-184* is abundantly expressed in cornea and lens. It was considered that miR-184 with this specific mutation fails to compete with another miRNA—*miR-205* for overlapping target sites on the 3′-UTR of two target genes,* INPPL1* (inositol polyphosphate phosphatase-like 1) and* ITGB4* (integrin beta 4). These two genes are involved in corneal healing after injury as the principal component of corneal basal epithelial hemidesmosomes [94]. The same mutation in* miR-184* has been replicated in other KC patients with congenital cataracts [96, 201]. Two additional mutations (r.8c>a and r.3a>g) were reported in sporadic KC patients with very low frequency (2 in 780 patients) [97]. These two sporadic KC patients did not have congenital cataracts. These two mutations may have incomplete or reduced penetrance in the studied families. However, we did not find any mutations in over 140 KC patients from Saudi Arabia (unpublished data). All these indicate that mutations in* miR-184* only account for a relative small number of KC patients or that miR-184 contributes to the causal of congenital cataract instead of KC. The identification of* miR-184* in KC patients suggests that regulatory variants may directly impact transcriptional activity of key target genes in cornea development and maintenance. More research will be necessary to study whether miR-184 may regulate the expression of other KC candidate genes.

Chr13q32 was originally identified to be linked with familial KC in Ecuadorian families, under an autosomal dominant model [90]. Mutation screening of 8 candidate genes in this region identified a potential mutation c.2262A>C (p. Gln754His) in* DOCK9* (dedicator of cytokinesis 9) in a large Ecuadorian KC family [91].* DOCK9* (OMIM 607325) encodes a member of the DOCK protein family with GTP/GDP exchange factor activity that specifically activates G-protein CDC42 [202].* DOCK9* is expressed in human cornea [91]. However, it still requires to be replicated in other KC families and patients [92] as well as functional work of the reported mutation in cornea.

### 4.2. Genome-Wide Association Studies

Genome-wide association studies (GWAS) examine several hundred thousand to over a million SNPs in hundreds to thousands of individuals using high throughput DNA genotyping technology [203]. GWAS has been shown to be very powerful to identify the genetic factors of many complex traits and diseases, including central corneal thickness (CCT) and KC. A number of GWAS reported the association of CCT with sequence variants near or within many genes, including* ZNF469*,* COL5A1*,* RXRA-COL5A1*,* COL8A2*,* AKAP13*,* AVGR8*,* FOXO1*,* FNDC3B*,* TJP1*,* NR3C2*,* LRRK1*,* FDF9-SGCG*,* LCN12-PTGDS*,* ADAMTS6*,* CHSY1*,* HS3ST3B1-PMP22*,* GLT8D2*,* SMAD3*,* VKORC1L1*,* COL4A3*,* FAM46A-IBTK*,* LPAR1*,* ARID5B*,* TBL1XR1-KCNMB2*,* ARHGAP20-POU2AF1*,* C7ORF42*,* MPDZ-NF1B*,* USP37*,* GPR15*, and* TIPARP* [204–208]. Two CCT-associated genomic regions* FOXO1* and* FNDC3B* have been associated with KC risk [207]. These genetic discoveries implicate the role of the collagen and extracellular matrix pathways in the regulation of CCT [207] and potentially KC. Recently, two studies identified that missense variants in* ZNF469* have been identified in 12.5% and 23.3% of sporadic KC patients in UK/Switzerland and New Zealand, respectively [209, 210], indicating the potential role of* ZNF469* in the development of KC. However, more replicative sequencing and further functional studies will need to determine the relative role of* ZNF469* in the pathogenesis of KC. Recently, our group has identified several genomic deletions in familial KC patients in several CCT-associated regions, including* RXRA-COL5A1* and* HS3ST3B1-PMP22*, as well as a refractive error-associated region of* GRIA4* [211]. The genetic variants in* ZNF469* and genomic deletions in these genes indicate the potential contributions of these CCT-associated genes in the pathogenesis of KC.

The first GWAS with KC was reported by Li et al. in 2011 in a Caucasian population of 222 patients and 3324 controls [212]. Although no genome-wide significant associations (*P* value < 5 × 10^−8^) were identified, a suggestive association (*P* value 1.6 × 10^−7^) was reported with a genomic region located near the* RAB3GAP1* (RAB3 GTPase activating protein subunit 1 (catalytic)) gene on chromosome 2q21.3. This association has been replicated in a separate study by Bae et al. [213], suggesting the genetic contribution of this region to KC susceptibility. RAB3GAP1 is involved in regulation of RAB3 activity by forming a heterodimer with RAB3GAP2 to convert active RAB3-GTP to the inactive form RAB3-GDP [214]. Interestingly, mutations in* RAB3GAP1* are associated with Warburg Micro Syndrome, a rare autosomal recessive syndrome with ocular and neurodevelopmental defects, such as microphthalmos, microcornea, congenital cataracts, and optic atrophy [214–217].

The second GWAS with KC was followed by Burdon et al. in a population of patients from Australia using pooled DNA from 97 KC patients and 216 controls [218]. While no variants reached genome-wide significance, the most significant association (9.9 × 10^−7^) was located upstream of the* HGF* (hepatocyte growth factor) gene. The specific variant was also associated with serum HGF level in normal individuals [218]. This association has been independently replicated by Sahebjada et al. [219]. HGF regulates cell growth, cell motility, and morphogenesis by activating a tyrosine signalling cascade [220]. The genomic region of* HGF* has been associated with refractive error in several populations including Han Chinese and Caucasians [221–223]. The association of* HGF* with KC suggests the potential involvement of HGF-related inflammatory pathways.

### 4.3. Candidate Genes

A large number of candidate genes have been studied in relation to KC pathogenesis. We will focus on two main candidate genes, visual system homeobox 1 (*VSX1*) and superoxide dismutase 1 (*SOD1*).* VSX1* is located within a linkage locus for a corneal dystrophy called posterior polymorphous dystrophy (PPCD) [224–226], which has been associated with KC [227–233]. Since PPCD and KC have similar corneal curvature and the involvement of posterior surface of cornea, specifically Descemet's membrane, PPCD and KC might be linked due to poor case definition. In 2002* VSX1* mutations were first reported in PPCD and KC patients [234], in which two mutations (R166W and L159 M) were originally identified in KC patients.* VSX1* encodes a pair-like homeodomain protein which binds to the core of the locus control region of the red and green visual pigment gene cluster and may regulate expression of the cone opsin genes during embryonic development [235, 236]. It is expressed in several ocular tissues including the retina [224, 226, 234]. The expression of* VSX1* in human or mouse cornea remains unclear since many studies did not confirm the expression in cornea [234, 236, 237]. Mouse models with the loss of* VSX1* function did not show cornea-related phenotypes [235]. Since the original report in 2002, many studies have examined the potential mutations of* VSX1* in KC patients [90, 238–255]. Most of the identified variants are polymorphic [199]. It remains unclear whether* VSX1* mutations contribute to the pathogenesis of KC [37, 162, 256]. It is possible that mutations in* VSX1* only affect a very small percentage of KC patients, which is consistent with the concept of genetic heterogeneity of KC. It is also more possible that* VSX1* may not play a significant role in the pathogenesis of KC. We recommend future research efforts focus in the identification of novel genetic factors in KC.

*SOD1* encodes a major cytoplasmic antioxidant enzyme that metabolizes superoxide radicals and provides a defence against oxygen toxicity [257]. Mutations in* SOD1* have been implicated in familial amyotrophic lateral sclerosis (ALS) [257, 258]. However, no corneal phenotypes have been reported in ALS patients. To date, it is widely accepted that oxidative stress plays a critical role in the progression of KC [37, 240]. An accumulation of cytotoxic by-products, mitochondrial DNA damage, and high levels of oxidative stress in KC-affected corneas [259–262] have been reported.* SOD1* has been selected as a candidate gene and examined in many KC-related studies [239, 249, 255, 263–265]. However, no mutations in* SOD1* have been identified in KC patients. It remains undetermined whether* SOD1* plays a role in the pathogenesis of KC.

### 4.4. Future Direction

Recent development in genome technology has enabled the application of novel and high throughput genetic approaches in ocular genetics research. Among these technologies, whole exome or genome sequencing will be very powerful in the identification of causal mutations in multiplex families with KC [266–268]. Many research laboratories around the world, including our group, have applied the whole exome sequencing to identify causal mutations in multiplex KC families. Previously identified linkage region will be tremendously helpful to assist the interpretation of exome or genome sequencing data. As discussed earlier, the genetic heterozygosity of KC may prevent a single research group from identifying and replicating novel genetic mutations. It will be necessary for different KC research groups to collaborate with each other, by sharing DNA samples and phenotype data. A genetics research consortium may be one of the approaches. The integration of next generation sequencing has recently led to the identification of* miR-184* mutations in KC patients. We expect to see more peer-reviewed reports using next generation sequencing in the near future. At the same time, in comparison to GWAS studies with small sample size, GWAS approach with large number of cases and controls in different ethnic groups will greatly improve the chances of avoiding type I errors and will continue to identify novel genomic variants that are associated with KC and cornea-relative phenotypes. In addition, gene expression profile in normal and diseased human cornea will provide further information to help narrow down the list of potential causal genes.

## 5. Conclusion

In summary, KC is the most common ectatic disorder of cornea with the onset of puberty. It affects both genders and all ethnic groups worldwide. Both environmental and genetic factors contribute to the pathogenesis of KC. Significant achievements have been made in the understanding of its epidemiology and etiology. Newly developed genetic technologies including whole exome or genome sequencing and genome-wide association technologies have promoted and will continue to improve our knowledge on the pathogenesis of KC. This knowledge will eventually lead to future development of improved early diagnostics, targeted therapeutics, and potential prognosis.

## Figures and Tables

**Table 1:** Hospital/clinic based epidemiological studies of KC.

Author	Location	Age in years	Sample size	Incidence/100,000	Prevalence/100,000	Method
Tanabe et al. (1985) [58]	Muroran, Japan	10–60	2601-P		9	Keratometry
Kennedy et al. (1986) [15]	Minnesota, USA	12–77	64-P	2	54.5	Keratometry + retinoscopy
Ihalainen (1986) [59]	Finland	15–70	294-P	1.5	30	Keratometry + retinoscopy
Gorskova and Sevost'ianov (1998) [60]	Urals, Russia				0.2–0.4	Keratometry
Pearson et al. (2000) [30]	Midlands, UK	10–44	382-P	4.5-W 19.6-A	57 229	Keratometry + retinoscopy
Ota et al. (2002) [28]	Tokyo, Japan		325-P	9		Keratometry?
Georgiou et al. (2004) [25]	Yorkshire, UK		74-P	3.3-W 25-A		Clinical examination
Assiri et al. (2005) [61]	Asir, Saudi Arabia	8–28	125-P	20		Keratometry
Nielsen et al. (2007) [62]	Denmark		NA	1.3	86	Clinical indices + topography
Ljubic (2009) [63]	Skope, Macedonia		2254		6.8	Keratometry
Ziaei et al. (2012) [64]	Yazd, Iran	25.7 ± 9	536	22.3 (221)		Topography

A, Asian (Indian, Pakistani, and Bangladeshi); W, white; P, patient; NA, not available.

**Table 2:** Population-based epidemiological studies of KC.

Author	Location	Age in years (mean)	Sample size	Prevalence/100,000 (cases)	Method	Sampling method
Hofstetter (1959) [65]	Indianapolis, USA	1–79	13345	120 (16)	Placido disc^Ψ^	Rural volunteers
Santiago et al. (1995) [66]	France	18–22	670	1190	Topography	Army recruits
Jonas et al. (2009) [33]	Maharashtra, India	>30 (49.4 ± 13.4)	4667	2300 (128)	Keratometry^Ψ^	Rural volunteers (8 villages)
Millodot et al. (2011) [27]	Jerusalem, Israel	18–54 (24.4 ± 5.7)	981	2340 (23)	Topography	Urban volunteers (1 college)
Waked et al. (2012) [67]	Beirut, Lebanon	22–26	92	3300 (3)	Topography	Urban volunteers (1 college)
Xu et al. (2012) [68]	Beijing, China	50–93 (64.2 ± 9.8)	3166	900 (27)	Optical low coherence reflectometry^Ψ^	Rural + urban volunteers
Hashemi et al. (2013) [69]	Shahrud, Iran	50.83 ± 0.12	4592	760 (35)	Topography	Urban volunteers from random cluster
Hashemi et al. (2013) [70]	Tehran, Iran	14–81 (40.8 ± 17.1)	426	3300 (14)	Topography	Urban volunteers (stratified cluster)
Shneor et al. (2014) [41]	Haifa, Israel	18–60 (25.05 ± 8.83)	314	3180 (10)	Topography	Urban volunteers (1 college)
Hashemi et al. (2014) [71]	Mashhad, Iran	20–34 (26.1 ± 2.3)	1073	2500 (26)	Topography	Urban volunteers (stratified cluster in 1 university)

^Ψ^The methods for detecting KC used in these studies are now considered inadequate and the results should be interpreted with caution.

**Table 3:** Percentage of allergy, asthma, and eczema in KC patients from several studies.

Study	Year	Allergy	Asthma	Eczema
Copeman [72]	1965	27		32
Karseras and Ruben [73]	1976	34.6	34.6	18.6
Rahi et al. [74]	1977	15	3	2
Gasset et al. [75]	1978	35.7	17.9	8.2
Swann and Waldron [76]	1986	42.2	15.8	12.3
Ihalainen [59]	1986	35	8	24
Harrison et al. [77]	1989	37.3	28.4	31.3
Tuft et al. [10]	1994	35.2	25.2	19.9
Zadnik et al. [39]	1998	53	14.9	8.4
Owens and Gamble [29]	2003	57	34	30
Mcmonnies and Boneham [78]	2003	39		
Georgiou et al. [25]	2004	20 W, 9 A	38 W, 18 A	14 W, 7 A
Assiri et al. [61]	2005	39.2	5.6	8
Weed et al. [79]	2008	30	23	14
Nemet et al. [80]	2010	17.6	8.2	
Jordan et al. [11]	2011	25.5	26.2	22.4
Khor et al. [81]	2011	1.8	26	18.4
Shneor et al. [82]	2013	34.4	13.2	6.6

A, Asian; W, white.

**Table 4:** List of the identified genomic loci through linkage studies.

Population	Location	Mode of inheritance	Gene	Reference
Australian	1p36.23-36.21	Autosomal dominant		[83]
Ecuadorian	2q13-q14.3	Autosomal dominant		[84]
European, Arabic, Caribbean African	2p24			[85]
Italian	3p14-q13	Autosomal dominant		[86]
Caucasian, Southern Italian	5q14.3-q.21.1	Autosomal dominant		[87, 88]
Caucasian, Hispanic	5q23.2			[89]
Southern Italian	5q32-q33			[87]
Australian	8q13.1-q21.11	Autosomal dominant		[83]
Caucasian, Hispanic	9q34			[89]
Ecuadorian	13q32	Autosomal dominant	DOCK9	[90–92]
Southern Italian	14q11.2			[87]
Caucasian, Hispanic	14q11.2			[89]
Multiethnic	14q24.3			[93]
Southern Italian	15q2.32			[87]
Northern Irish	15q22.33-24.2	Autosomal dominant	miR-184	[94–97]
Finnish	16q22.3-q23.1	Autosomal dominant		[98]
Pakistani	17p13	Autosomal recessive		[99]
Ecuadorian	20p13-p12.2			[84]
Australian, Tasmania	20q12	Autosomal dominant		[100]
